# Systems Genetic Analysis of Osteoblast-Lineage Cells

**DOI:** 10.1371/journal.pgen.1003150

**Published:** 2012-12-27

**Authors:** Gina Calabrese, Brian J. Bennett, Luz Orozco, Hyun M. Kang, Eleazar Eskin, Carlos Dombret, Olivier De Backer, Aldons J. Lusis, Charles R. Farber

**Affiliations:** 1 Center for Public Health Genomics, University of Virginia, Charlottesville, Virginia, United States of America; 2 Department of Genetics, University of North Carolina at Chapel Hill, Chapel Hill, North Carolina, United States of America; 3 Department of Medicine, David Geffen School of Medicine, University of California Los Angeles, Los Angeles, California, United States of America; 4 Department of Biostatistics, University of Michigan, Ann Arbor, Michigan, United States of America; 5 Department of Computer Science, University of California Los Angeles, Los Angeles, California, United States of America; 6 Unité de Recherche en Physiologie Moléculaire (URPHYM), Namur Research Institute for Life Sciences (NARILIS), FUNDP School of Medicine, University of Namur, Namur, Belgium; 7 Department of Human Genetics, David Geffen School of Medicine, University of California Los Angeles, Los Angeles, California, United States of America; 8 Department of Microbiology, Immunology, and Molecular Genetics, University of California Los Angeles, Los Angeles, California, United States of America; 9 Department of Medicine, Division of Cardiovascular Medicine, University of Virginia, Charlottesville, Virginia, United States of America; 10 Department of Biochemistry and Molecular Genetics, University of Virginia, Charlottesville, Virginia, United States of America; National Institute of Genetics, Japan

## Abstract

The osteoblast-lineage consists of cells at various stages of maturation that are essential for skeletal development, growth, and maintenance. Over the past decade, many of the signaling cascades that regulate this lineage have been elucidated; however, little is known of the networks that coordinate, modulate, and transmit these signals. Here, we identify a gene network specific to the osteoblast-lineage through the reconstruction of a bone co-expression network using microarray profiles collected on 96 Hybrid Mouse Diversity Panel (HMDP) inbred strains. Of the 21 modules that comprised the bone network, module 9 (M9) contained genes that were highly correlated with prototypical osteoblast maker genes and were more highly expressed in osteoblasts relative to other bone cells. In addition, the M9 contained many of the key genes that define the osteoblast-lineage, which together suggested that it was specific to this lineage. To use the M9 to identify novel osteoblast genes and highlight its biological relevance, we knocked-down the expression of its two most connected “hub” genes, *Maged1* and *Pard6g*. Their perturbation altered both osteoblast proliferation and differentiation. Furthermore, we demonstrated the mice deficient in *Maged1* had decreased bone mineral density (BMD). It was also discovered that a local expression quantitative trait locus (eQTL) regulating the *Wnt* signaling antagonist *Sfrp1* was a key driver of the M9. We also show that the M9 is associated with BMD in the HMDP and is enriched for genes implicated in the regulation of human BMD through genome-wide association studies. In conclusion, we have identified a physiologically relevant gene network and used it to discover novel genes and regulatory mechanisms involved in the function of osteoblast-lineage cells. Our results highlight the power of harnessing natural genetic variation to generate co-expression networks that can be used to gain insight into the function of specific cell-types.

## Author Summary

The osteoblast-lineage consists of a range of cells from osteogenic precursors that mature into bone-forming osteoblasts to osteocytes that are entombed in bone. Each cell in the lineage serves a number of distinct and critical roles in the growth and maintenance of the skeleton, as well as many extra-skeletal functions. Over the last decade, many of the major regulatory pathways governing the differentiation and activity of these cells have been discovered. In contrast, little is known regarding the composition or function of gene networks within the lineage. The goal of this study was to increase our understanding of how genes are organized into networks in osteoblasts. Towards this goal, we used microarray gene expression profiles from bone to identify a group of genes that formed a network specific to the osteoblast-lineage. We used the knowledge of this network to identify novel genes that are important for regulating various aspects of osteoblast function. These data improve our understanding of the gene networks operative in cells of the osteoblast-lineage.

## Introduction

The osteoblast-lineage consists of a spectrum of cells beginning with osteoprogenitors derived from mesenchymal stem cells that then differentiate to form mature bone-forming osteoblasts and bone-lining cells. The final stage in the life-cycle of the lineage occurs when a subset of mature osteoblasts become entombed in bone as mechanosensitive osteocytes [1]. As the only known bone-forming cell, osteoblasts are essential for skeletal development, growth and maintenance [1]. In addition to their critical role in the skeleton, osteoblast-lineage cell have been shown to be important for other physiological systems. Osteoprogenitors can support and modulate erythropoiesis [2] and mature osteoblasts are responsible for many of the endocrine functions of bone, including the regulation of energy expenditure [3]–[5] and male fertility [6]. Furthermore, osteocytes play important roles in mineral metabolism [7] and bone resorption [8], [9]. Therefore, the development of a more comprehensive understanding of the molecular networks operative in osteoblast-lineage cells will have important implications not only for osteoporosis, but many other common complex diseases.

Genetic, molecular and biochemical approaches have been used over the last decade to identify many of the key genes that are required for osteoblast progenitor commitment, proliferation, differentiation and apoptosis as well as mature osteoblast and osteocyte activity [1]. An example of this has been the discovery that the *Wnt* signaling pathway plays a central role in many functional aspects of the osteoblast lineage [10]. However, these investigations have been reductionist in nature and therefore have not provided information on how key signaling genes interact in complex cellular networks, which is critical to fully understand the molecular mechanisms that underlie cellular processes and disease. In many cases, the insight gained regarding how genes interact in networks goes well beyond what can be learned about a process using only traditional approaches [11].

Systems genetics is an emerging approach that provides a systems-level perspective of the role of genetic variation in cell function and disease [12]. Systems genetics relies on the principles and methods of systems biology, but focuses on determining how naturally occurring genetic variation perturbs cellular phenotypes [13]. The foundation of systems genetics is a suite of analytical approaches that include genome-wide association, expression quantitative trait locus (eQTL) discovery, causality modeling and network analysis [14].

One of the most powerful systems genetics tools is network analysis. Biological networks can be based on many different types of interactions [15], such as genetic, protein-protein and transcription factor binding. However, the most common networks used in systems genetics research are based on co-expression. In a systems genetics context, co-expression networks are generated using global expression data collected across many genetically unique individuals [16]. The patterns of gene expression that result from each unique set of genetic perturbations are used to quantify correlational relationships among genes on a genome-wide scale. Co-expression networks typically display two important behaviors, i) they are modular, with distinct modules representing dense clusters of genes that are highly co-expressed and ii) the co-expression modules are often enriched for genes that share similar functions [17]. Because modules contain functionally similar gene sets, they can be used to extract many pieces of information about a system. For instance, a number of studies have shown that summarized measures of module behavior often correlate with complex processes or disease phenotypes [18]–[20]. The identification of such modules provides a list of genes and pathways that likely play a role in the process or disease. In addition, genes within a module can be organized by connectivity. Highly connected genes are called “hubs” and in a co-expression network hub genes are those that are the most strongly correlated with the largest number of other module genes [21]. Importantly, a number of studies have found that connectivity correlates with biologically relevant properties. For example, hubs in yeast networks have been found to be more likely essential for growth than non-hub genes [22] and connectivity was found to be predictive of survival in a human brain co-expression module associated with glioblastoma [23]. Recently, we demonstrated that in a co-expression module associated with Bone Mineral Density (BMD) in humans, hub genes were more likely to be genetically associated with BMD than non-hub genes [24]. We have also shown that hubs within a chrondrocyte co-expression network play key roles in chrondrocyte differentiation [20].

In this study, we reconstructed a bone co-expression network using Weighted Gene Co-expression Network Analysis (WGCNA) and gene expression microarray profiles from femur samples collected from 96 Hybrid Mouse Diversity Panel (HMDP) strains. The resulting network was used to identify a core module of genes (module 9; M9) specific to cells of the osteoblast-lineage. We then demonstrated that the top two M9 hub genes were regulators of osteoblast function and one was a regulator of BMD *in vivo*. In addition, we showed that a local eQTL regulating the expression of secreted frizzled-related protein 1 (*Sfrp1*), orchestrated the transcriptional behavior of the M9. Notably, *Sfrp1* is an antagonist of *Wnt* signaling, which is a major pro-osteoblastic signal, and *Sfrp1* transgenic and knockout mice display alterations in BMD and osteoblast functions [25], [26]. We further demonstrated the physiologically relevant nature of the M9 by identifying a strong non-linear relationship between the M9 and BMD in the HMDP and that the M9 is enriched for genes implicated in the regulation of human BMD through genome-wide association studies. In summary, our results begin to clarify the composition and role of cellular networks in the osteoblast cell lineage.

## Results

### Generation of a WGCNA network for bone in the HMDP

A genome-wide co-expression network for bone was constructed by applying WGCNA to microarray gene expression profiles from femur samples collected on 96 HMDP strains [27], [28]. The network was generated using all 45,719 expression probes (representing 30,264 unique genes) present on the Illumina Mouse WG6 microarrays. Of the total, 13,759 probes (10,968 unique genes) were assigned to one of 21 co-expression modules (Table 1). All other probes were not assigned to a module. Of the 21 modules, all but module 15 was enriched for genes belonging to similar genome ontology (GO) or Kyoto Encyclopedia of Genes and Genomes (KEGG) functional groupings (Table 1). Lists of module assignments for all genes and all significant (FDR≤0.05) functional enrichments are provided in File S1 and File S2.

**Table 1:** Number of genes and most significant functional enrichment for each of the 21 bone network modules.

Module	No. Probes	No. Unique Genes	Term	Percent^a^	Fold Enrichment^b^	FDR
1	2835	2504	GO:0007049∼cell cycle	7.4	2.5	4.0×10^−30^
2	2551	2204	GO:0005739∼mitochondrion	16.6	2.5	1.1×10^−64^
3	1752	1715	GO:0007608∼sensory perception of smell	9.5	2.0	3.9×10^−14^
4	1107	981	GO:0044265∼cellular macromolecule catabolic process	8.4	2.7	2.3×10^−14^
5	1106	1077	mmu03010:Ribosome	2.8	6.4	5.1×10^−14^
6	1017	910	GO:0005578∼proteinaceous extracellular matrix	6.4	4.7	4.4×10^−20^
7	724	615	GO:0055114∼oxidation reduction	16.6	4.7	7.2×10^−38^
8	452	425	GO:0000278∼mitotic cell cycle	4.4	4.4	6.3×10^−5^
9	400	354	GO:0031012∼extracellular matrix	10.6	6.0	1.9×10^−16^
10	320	301	GO:0005739∼mitochondrion	17.4	2.5	2.5×10^−8^
11	279	278	GO:0031226∼intrinsic to plasma membrane	6.0	2.7	3.7×10^−2^
12	237	219	GO:0006814∼sodium ion transport	9.4	14.9	7.7×10^−16^
13	227	199	GO:0050817∼coagulation	7.1	23.4	3.4×10^−12^
14	174	171	GO:0005739∼mitochondrion	32.9	4.5	3.3×10^−22^
15	98	96	NS^c^	NS	NS	NS
16	95	94	GO:0015931∼nucleobase, nucleoside, nucleotide and nucleic acid transport	4.8	13.1	4.9×10^−2^
17	88	76	mmu04142:Lysosome	14.7	17.7	1.5×10^−9^
18	80	68	GO:0015629∼actin cytoskeleton	14.1	12.5	4.8×10^−6^
19	78	74	GO:0031981∼nuclear lumen	17.3	4.1	9.7×10^−3^
20	75	69	GO:0001944∼vasculature development	16.4	11.5	3.6×10^−7^
21	64	54	GO:0006955∼immune response	36.2	14.4	3.0×10^−14^

a Percent = The percentage of module genes belonging to the listed GO or KEGG term.

b Fold Enrichment = The ratio of the percentage of module genes with the listed GO or KEGG term relative to the percentage of genes belonging to that term across the genome.

c NS = Not significant. There were no significant enrichments for module 15.

### Module 9 is specific to the osteoblast-lineage

**Figure 1:**
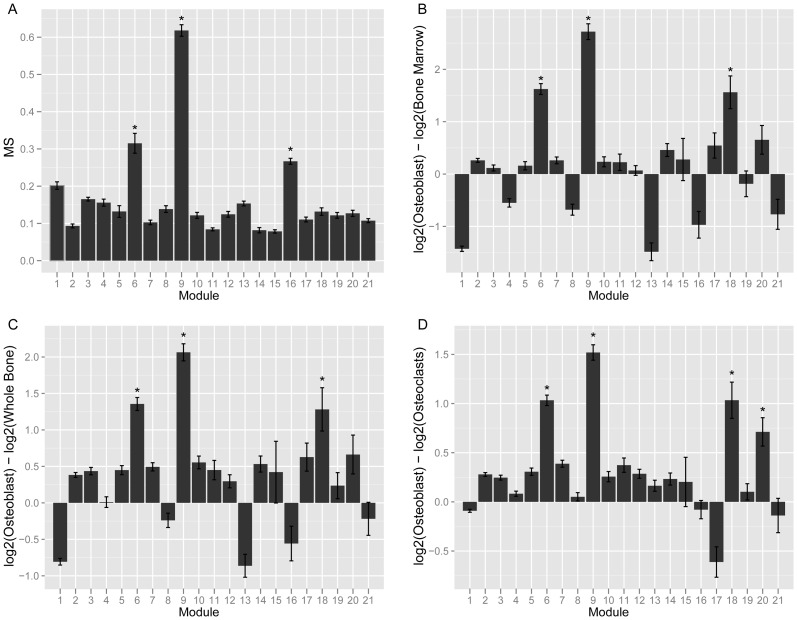
Module 9 is a co-expression network specific to cells of the osteoblast-lineage. (A). Mean MS score for each of the 21 network modules. (B) Mean module gene expression in osteoblasts relative to bone marrow. (C) Mean module gene expression in osteoblasts relative to whole bone. (D) Mean module gene expression in osteoblasts relative to osteoclasts. In panels (B–D) expression is presented as the mean log2 expression for each gene in a module in osteoblasts minus log2 expression in the second sample. *Bonferroni adjusted P<0.002.

Given that bone is a heterogeneous tissue, we expected that a subset of modules from the network would represent cell-type specific networks. Therefore, we next set out to identify the co-expression module that was the most relevant for the function of osteoblast-lineage cells. We calculated two metrics, the Gene Significance (GS) and Module Significance (MS) scores. The GS for each network gene was defined as the absolute value of the correlation between its expression and the eigengene summarizing the expression of a group of nine (*Col1a1*, *Col1a2*, *Akp2*, *Bglap1*, *Sp7*, *Ibsp*, *Sost*, *Mmp13*, *Tnfrsf11b*, *Dmp1* and *Phex*) osteoblast/osteocyte markers. These genes were selected prior to the network analysis based on mining the literature for widely used markers of osteoblasts and osteocytes. The MS was defined as the mean GS for each module. In bone, the above marker genes are preferentially (or exclusively) expressed in cells of the osteoblast-lineage; therefore, a module with a high MS would be expected to represent a sub-network of genes that function specifically in these cells. Of the 21 modules, module 9 (M9) had the highest MS (MS = 0.62±0.02; P<0.002). The next highest MS scores were observed for module 6 (MS = 0.32±0.02; P<0.002) and module 16 (MS = 0.27±0.01; P<0.002) (Figure 1A). All other modules had MS scores at or below 0.20 (P>0.002).

We also expected that the most relevant module would contain genes more highly expressed in osteoblasts than other bone cells. To evaluate the expression patterns of all network genes we utilized an independent set of microarray data which surveyed global gene expression in primary osteoblasts (at 5, 14 and 21 days of *in vitro* differentiation), primary osteoclasts, bone marrow and whole bone [29]. M9 genes were over 4-fold (P<0.05) more highly expressed in osteoblasts than whole bone or bone marrow and 2.8-fold (P<0.05) more highly expressed in osteoblasts than osteoclasts (Figure 1B–1D).

The M9 was significantly (FDR≤0.05) enriched for 53 GO and KEGG pathway terms (File S1). These included terms such as “extracellular matrix” (FDR = 1.9×10^−16^), “collagen” (FDR = 4.9×10^−7^), “ossification” (FDR = 3.0×10^−5^), “bone development” (FDR = 8.6×10^−5^), “skeletal system development” (FDR = 4.9×10^−4^), “Wnt receptor signaling pathway” (FDR = 1.2×10^−3^) and “regulation of bone mineralization” (FDR = 3.7×10^−2^), which are relevant to osteoblasts. Additionally, we found that the M9 was enriched (Fisher's exact test P = 2.8×10^−8^) in genes belonging to a list of 254 that were members of 11 GO terms containing the term “osteoblast” (such as “regulation of osteoblast differentiation” (GO:0045667) and “osteoblast proliferation” (GO:0033687)) or their perturbation in a mouse model has been observed to affect osteoblast function. Lastly, the M9 contained many known genes that, in bone, are specific to or define the osteoblast-lineage. Examples include *Akp2* (alkaline phosphatase), *Bglap1* (bone gamma carboxyglutamate protein; osteoclacin), *Cd276* (CD276 molecule), *Col1a1* (collagen, type I, alpha 1), *Col1a2* (collagen, type I, alpha 2), *Lrp5* (low density lipoprotein receptor-related protein 5), *Mmp2* (matrix metallopeptidase 2), *Pthr1* (parathyroid hormone receptor 1), *Sp7* (Sp7 transcription factor 7), *Tnfrsf11b* (tumor necrosis factor receptor superfamily, member 11b) and *Wnt5a* (wingless-related MMTV integration site 5A). Together, these data indicate that M9 represents a core gene network specific to cells of osteoblast-lineage.

### M9 connectivity is correlated with GS

**Figure 2:**
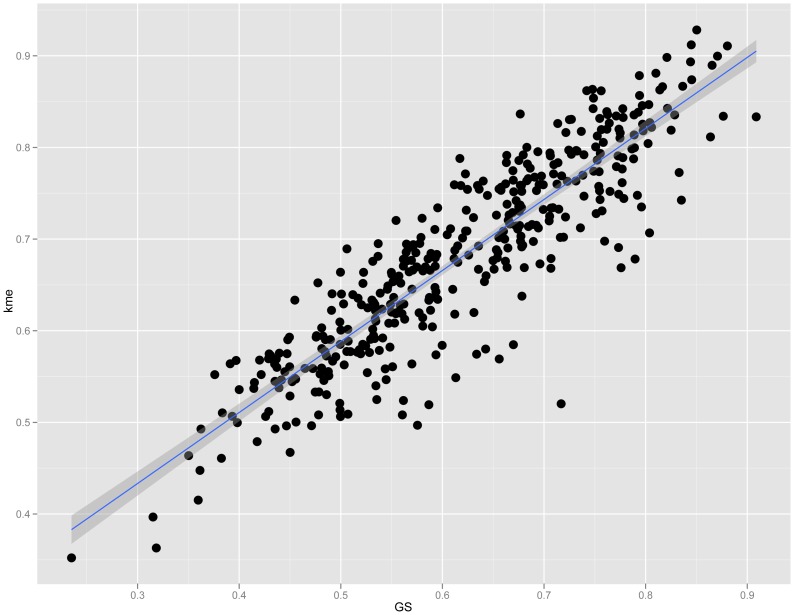
Connectivity is strongly correlated with GS in module 9. Plot showing the correlation (r = 0.89, P = 7.7×10^−141^) between connectivity (kme) and GS among module 9 genes.

To characterize the M9 network we first determined if M9 topology was important for the function of osteoblast lineage cells. We began by evaluating connectivity, a parameter of network topology. Recently, a number of studies have shown that highly connected “hub” genes tend to play critical roles in module organization [19], [24]. We defined connectivity (kme) for each gene as the correlation between its expression and its module eigengene [21]. Importantly, kme is a property inherent to each gene and would not be expected to correlate with other individual gene metrics (such as GS), unless the organization of the module was an important property of the system. A strong positive correlation was observed between M9 gene kme and GS (r = 0.89, P = 7.7×10^−141^) (Figure 2), indicating that the more highly connected an M9 gene, the more closely its expression resembles that of a prototypical gene of the osteoblast lineage.

### M9 hub genes, *Maged1* and *Pard6g*, are novel regulators of osteoblast activity

**Figure 3:**
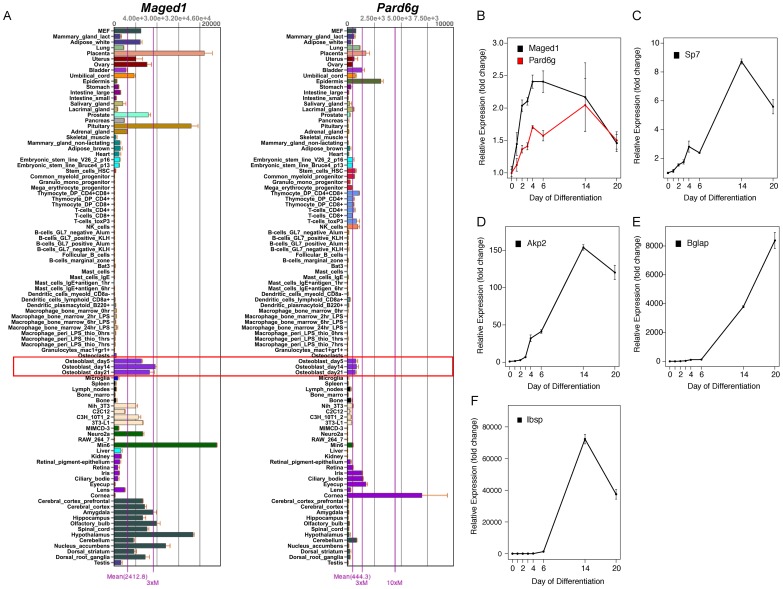
*Maged1* and *Pard6g* are expressed in osteoblasts. (A) *Maged1* and *Pard6g* are expressed in multiple mouse tissues and cell-lines including primary calvarial osteoblasts (outlined in red). The data are from a microarray experiment of tissues and cell lines (tissues and primary cell lines from C57BL6/J mice) and are the mean±SEM of three biological replicates [29]. Images were downloaded and modified from BioGPS (http://biogps.org/) [65]. The expression of (B) *Maged* and *Pard6g,* (C) *Sp7,* (D) *Akp2*, (E) *Bglap* and (F) *Ibsp* expression as a function of differentiation in primary calvarial osteoblasts (P = 0.03 for *Maged1* and *Pard6g* and P<0.001 for all other genes). The data represent mean±SEM (N = 4 at each timepoint).

The immediate implication of this finding is that we could use kme to determine if M9 hubs play a role in osteoblast function. The top 10 M9 hub genes are listed in Table 2. We focused on the two genes with the highest kme (Table 2), melanoma antigen, family D, 1 (*Maged1*) and par-6 partitioning defective 6 homolog gamma (C. elegans) (*Pard6g*). *Maged1* is a transcriptional co-activator that has been implicated in the regulation of myogenic differentiation [30], sexual behavior [31], obesity [31] and the transcriptional function of *Dlx5* [32], a positive regulator of osteoblast differentiation and bone mass [33], [34]. *Pard6g* is a homologue of the Par (partitioning defective) family of proteins that are involved in the regulation of cell polarity. We characterized the broad expression profiles of *Maged1* and *Pard6g* using microarray data from 96 mouse tissues and cell-types [29]. *Maged1* and *Pard6g* were expressed in multiple samples including primary calvarial osteoblasts (pcOBs) (Figure 3A). To confirm these data, the expression of both genes was measured during differentiation in an independent set of pcOBs. *Maged1* and *Pard6g* were differentially expressed (P = 0.03 for both genes) as a function of osteoblast differentiation (Figure 3B). *Maged1* expression increased rapidly after the induction of differentiation, peaked at day 6 and then decreased through day 20, possibly indicating a more important role for *Maged1* in early osteoblastogenesis. In contrast, *Pard6g* expression increased more slowly, peaking at day 14 and then decreasing by day 20. The expression of *Pard6g* was highly similar to established markers of osteoblast maturation (Figure 3C–3F), especially *Sp7*, *Akp2* and *Ibsp*.

**Table 2:** Ten most highly connected genes in the M9.

Gene	Description	Chr	Mb	kme	Role in Osteoblasts	Ref.
*Maged1*	melanoma antigen, family D, 1	X	91.8	0.93	Osteoblast proliferation and differentiation and BMD *in vivo*	This study
*Pard6g*	par-6 partitioning defective 6 homolog gamma (C. elegans)	18	80.3	0.91	Osteoblast proliferation and differentiation	This study
*Kdelr3*	KDEL (Lys-Asp-Glu-Leu) endoplasmic reticulum protein retention receptor 3	15	79.4	0.91	Unknown	NA
*Kdelr3* a	KDEL (Lys-Asp-Glu-Leu) endoplasmic reticulum protein retention receptor 3	15	79.4	0.90	Unknown	NA
*Rcn3*	reticulocalbin 3, EF-hand calcium binding domain	7	52.3	0.90	Downregulated in osteosarcoma	[66]
*C76566*	expressed sequence C76566	8	107.8	0.89	a.k.a B3gnt9-ps; Unknown	NA
*Akp2*	alkaline phosphatase 2, liver	4	137.3	0.89	Osteoblast marker; bone mineralization	[67]
*Col5a1*	procollagen, type V, alpha 1	2	27.9	0.88	Low abundance fibrillar collagen in bone	[68]
*Fkbp10*	FK506 binding protein 10	11	100.3	0.88	Mutations lead to Osteogenesis imperfecta, type VI	[69]
*Tmem119*	transmembrane protein 119	5	114.2	0.87	Osteoblast differentiation	[70]
*Gja1*	gap junction membrane channel protein alpha 1	10	56.1	0.87	Osteoblast differentiation	[71]

a The two different entries for Kdelr3 are from two separate microarray probes.

**Figure 4:**
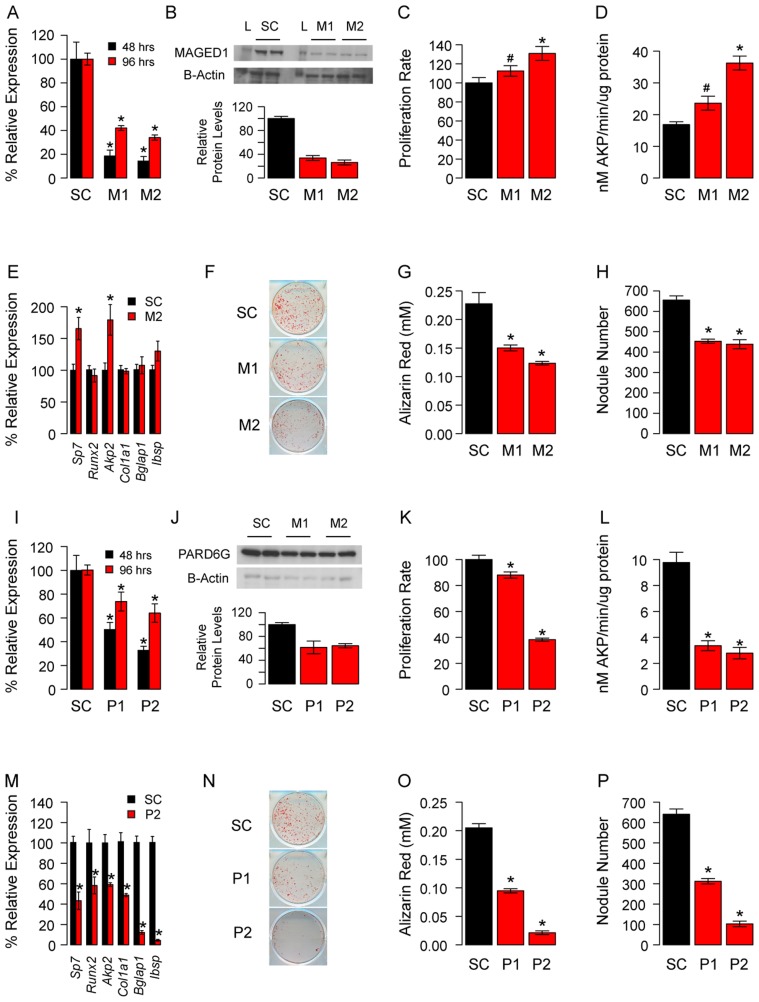
*Maged1* and *Pard6g* are novel regulators of osteoblast proliferation and differentiation. (A) The siRNAs M1 and M2 significantly reduced the levels of *Maged1* in undifferentiated pcOBs relative to a scrambled control (SC) at 48 and 96 hours post-differentiation. (B) This resulted in similarly decreased MAGED1 protein at 48 hours post-differentiation (L = protein ladder). (C) In undifferentiated pcOBs *Maged1* knockdown increased proliferation rate. (D) After four days of osteogenic differentiation *Maged1* knockdown increased alkaline phosphatase activity (E) and the expression of *Sp7* and *Akp2*. In contrast, at 14-days post-differentiation *Maged1* knockdown significantly decreased (F) mineralized nodule formation as determined by (G) Alizarin Red staining and (H) quantification of nodule number. (I) The siRNAs P1 and P2 significantly reduced the levels of *Pard6g* in undifferentiated pcOBs relative to a scrambled control (SC) at 48 and 96 hours post-differentiation. (J) This resulted in similarly decreased PARD6G protein at 96 hours post-differentiation. (K) In undifferentiated pcOBs *Pard6g* knockdown increased proliferation rate. (L) After four days of osteogenic differentiation *Pard6g* knockdown decreased alkaline phosphatase activity (M) and the expression of *Sp7*, *Runx2*, *Akp2*, *Col1a1*, *Bglap1* and *Ibsp*. (N) At 14-days after differentiation *Pard6g* knockdown decreased mineralized nodule formation as determined by (O) Alizarin Red staining and (P) quantification of nodule number. In all panels *P<0.05 and #P<0.10. The data represent mean±SEM (N = 4–6 independent experiment, except for Westerns (N = 2)).

We next determined the effects of *Maged1* and *Pard6g* knockdown on osteoblast proliferation and differentiation. Two independent siRNAs (M1 and M2 for *Maged1* and P1 and P2 for *Pard6g*) were used to target each gene. At 48 hours post-transfection in undifferentiated pcOBs, *Maged1* transcript levels were reduced to 18% and 14% of control in M1 and M2 transfected cells, respectively (P<0.05) (Figure 4A). At 96 hours post-transfection in these cells, *Maged1* knockdown was lower at 42% and 33% of control in M1 and M2 transfected cells, respectively (P<0.05) (Figure 4A). Knockdown at the transcript level resulted in similar reductions in MAGED1 protein levels 48 hours after transfection (Figure 4B). *Maged1* knockdown resulted in a 12% (P<0.10) and 31% (P<0.05) increase in proliferation rate in M1 and M2 transfected undifferentiated pcOBs, respectively (Figure 4C). In differentiating pcOBs (4 days), M1 and M2 treatment led to 40% (P<0.10) and 118% (P<0.05) increases in alkaline phosphatase activity (a marker of maturing osteoblasts), respectively (Figure 4D). We also observed 69% and 74% increases in the transcript levels of the osteoblastic genes *Sp7* and *Akp2,* respectively, in M2 treated cells (P<0.05) (Figure 4E). No differences were observed for the other osteoblast markers assayed (Figure 4E). Surprisingly, we observed a significant (P<0.05) decrease in the ability of M1 and M2 transfected osteoblasts to form mineralized nodules (a marker of mature osteoblast function) at 14 days post-differentiation (Figure 4F–4H). All the differences demonstrated a dose-dependent relationship with the extent of *Maged1* knockdown a the transcript level (the effect of M2>M1), suggesting that the effects were specific for the reduction in *Maged1* levels.

At 48 hours post-transfection in undifferentiated pcOBs, *Pard6g* transcript levels were reduced to 50% and 33% of control in P1 and P2 transfected cells, respectively (P<0.05) (Figure 4I). At 96 hours post-transfection, *Pard6g* knockdown was lower at 74% and 63% of control in P1 and P2 transfected cells, respectively (P<0.05) (Figure 4I). Knockdown at the transcript level resulted in similar reductions in PARD6G protein levels 48 hours after transfection (Figure 4J). *Pard6g* knockdown resulted in a 12% (P = 0.10) and 62% (P<0.05) decrease in proliferation rate in P1 and P2 transfected cells, respectively (Figure 4K). In differentiating pcOBs (4 days), P1 and P2 treatment led to 66% and 71% decreases (P<0.05) in alkaline phosphatase activity, respectively (Figure 4L). Additionally, 40% to 95% decreases (P<0.05) were seen in the levels of the osteoblast markers *Sp7, Runx2, Akp2, Col1a1, Bglap1* and *Ibsp* (Figure 4M). Consistent with these observations, there was a significant (P<0.05) impairment in the formation of mineralized nodules in P1 and P2 transfected cells (Figure 4N–4P). All the differences demonstrated a dose-dependent relationship with the extent of *Pard6g* knockdown at the transcript level (the effect of P2>P1), suggesting that the effects were specific for the reduction in *Pard6g* levels. These data indicate that the M9 hub genes, *Maged1* and *Pard6g*, are novel regulators of osteoblast activity.

### *Maged1*-deficient mice have decreased BMD

**Figure 5:**
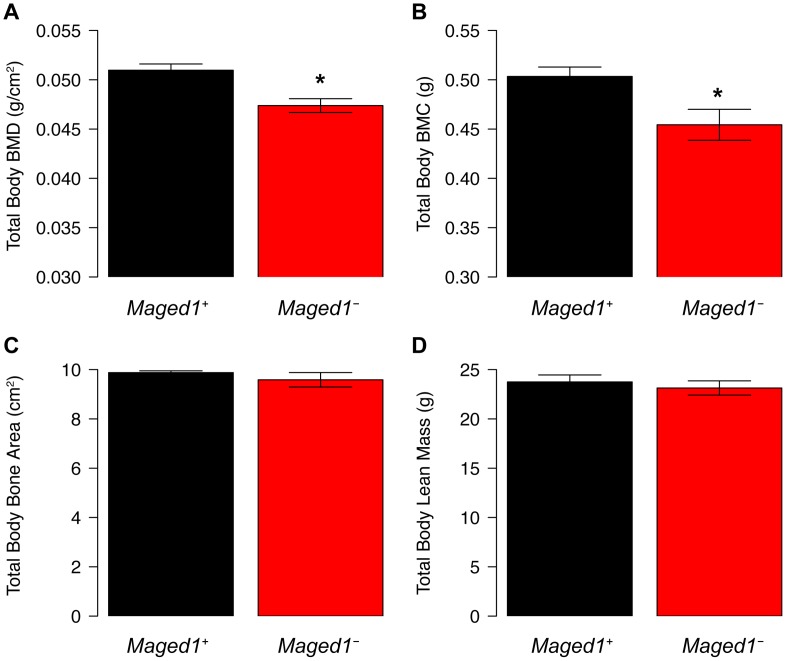
BMD is decreased in *Maged1*-deificient mice. (A) BMD and (B) BMC is decreased in *Maged1* ^−^ mice relative to wild-type littermates (*Maged1* ^+^). There is no difference in (C) bone area or (D) lean mass between genotypes. *P<0.05.

To further assess the physiological function of *Maged1*, we measured total body BMD in *Maged1* deficient mice (*Maged1* ^−^). In 18 week-old male *Maged1* ^−^ mice, we observed significantly (P<0.05) decreased BMD, relative to wild-type littermates (*Maged1* ^+^) (Figure 5A). This difference was primarily due to a decrease in bone mineral content (BMC) and not skeletal size (Figure 5B and 5C). Additionally, the difference was not a reflection of alterations in lean body mass that would alter BMD (Figure 5D).

### Secreted frizzled-related protein 1 (*Sfrp1*) is a regulator of the M9

We next sought to identify genetic loci responsible for the coordinate expression of M9 genes. Two strategies were employed for this analysis, the identification of expression QTL (eQTL) hotspots and genome-wide association (GWA). Based on previous studies [35], we anticipated that the identification of eQTL hotspots would have more statistical power than genome-wide association, but less mapping resolution. In contrast, genome-wide association would be less powerful, but would allow us to more precisely define the location of potential regulators. We choose *a priori* to focus only on regions that were implicated by both analyses, as these were the most likely to harbor true regulators.

**Figure 6:**
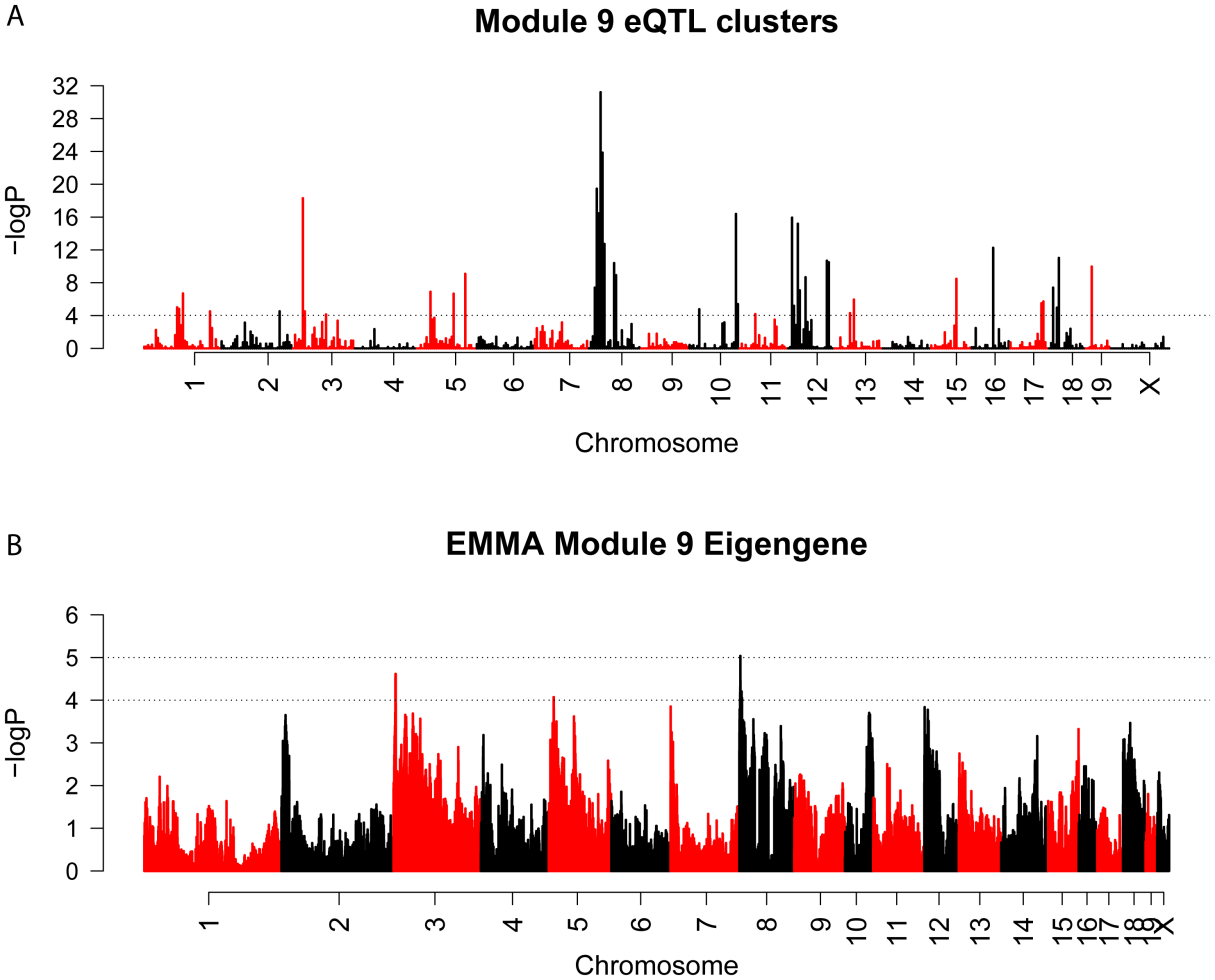
EQTL hotspot identification and genome-wide association identifies a regulator of M9 on Chromosome 8. (A) Genome-wide scan for regions associated with the expression of a large number of M9 genes. The dashed line is a genome-wide significance threshold of −log10(P = 9.4×10^−5^). (B) EMMA genome-wide association scan for the M9 eigengene.

We used the Efficient Mixed Model Algorithm (EMMA) [36] to perform GWA for all network genes. The number of M9 genes with eQTLs (−logP>4) in 5 Mbp bins across the genome was counted and compared to the frequency of eQTL for all other network genes. Several significant (Bonferroni corrected P<9.4×10^−5^) bins were identified, suggesting that the regulation of M9 gene expression was polygenic. The most prominent hotspots were located on Chrs. 8 (P = 6.1×10^−32^) and 3 (P = 5.1×10^−19^) (Figure 6A). We next used EMMA to directly identify associations for the M9 eigengene. In both cases, the top two hotspots/associations were concordant. The SNP (rs33030926, P = 9.0×10^−6^) that was the most strongly associated with the M9 eigengene was located on Chr. 8 at 24.587852 Mbp. (Figure 6B).

Both analyses provided strong evidence for the presence of a regulator of the M9 on Chr. 8 at ∼24.5 Mbp. It is possible that such a regulator influences M9 gene expression through a genetically regulated difference in its own expression and this would be detectable as a local eQTL. To determine if this was the case we identified all microarray probes mapping between 20 and 30 Mbp on Chr. 8. A total of 237 probes corresponding to 137 unique genes were located within the region. EMMA was used to perform genome-wide association for each probe [36]. We then selected the probe for each gene with the most significant local eQTL. A total of 15 genes were found to be regulated by significant (P≤3.6×10^−4^) local eQTL after correcting for multiple comparisons (Table 3). We would expect that the causal genes expression should be correlated with M9 gene expression. Thus, we calculated the proportion of M9 genes whose expression was correlated (r>|0.25|, nominal P<0.01) with the expression of each candidate regulator. Most of the 15 candidates showed little overlap (between 0 and 24%). However, the expression of *Sfrp1* correlated with 88.8% of M9 genes at a threshold of r>|0.25| (Table 3). *Sfrp1* was correlated with all 400 M9 probes if the threshold was reduced to r>|0.15|.

**Table 3:** *Sfrp1* is predicted to be a regulator of the M9.

Gene	Chr	Mbp	eSNP P	SNPs^a^	Overlap (%)^b^	ME r^c^	LEO.NB.AtoB^d^
*Nek3*	8	23.230919	1.2E-16	0	25.8	−0.27	−3.21
*Defcr15*	8	23.230919	3.4E-04	0	0.3	0.06	−4.75
*Defcr20*	8	23.381591	2.3E-04	1	2.3	0.05	−4.72
*AI316807*	8	23.643226	1.9E-08	0	6.8	−0.14	−4.28
*Mrps31*	8	23.993464	9.9E-09	4	23.3	−0.18	−4.04
*Ank1*	8	24.129746	2.5E-08	0	18.8	−0.23	−3.48
*Gins4*	8	24.316596	9.9E-07	0	17.0	−0.18	−4.78
*Golga7*	8	24.4321	5.9E-12	1	12.0	0.21	−3.72
***Sfrp1***	**8**	**24.587852**	**5.0E-11**	**0**	**88.8**	**0.53**	**2.8**
*Whsc1l1*	8	27.001571	1.6E-05	0	2.3	0.02	−4.54
*Hgsnat*	8	27.348861	1.7E-12	2	0.8	−0.07	−4.43
*Erlin2*	8	28.078367	2.7E-04	2	4.5	0.12	−4.85
*Prosc*	8	28.171596	1.4E-16	0	11.8	−0.09	−4.53
*Eif4ebp1*	8	28.371247	1.9E-14	0	9.5	−0.14	−4.48
*5430430B14Rik*	8	28.377596	1.9E-06	3	15.0	−0.24	−3.76

a SNPs = The number of SNPs (from dbSNP128) that overlap probes for each gene that may be giving rise to falsely significant eSNP. Note that the probe representing *Sfrp1* does not overlap with a known SNP.

b Overlap = The percentage of module 9 genes correlated with Sfrp1 at |r>0.25|.

c ME r = Pearson correlation between each candidates expression and the module 9 eigengene.

d LEO.NB.AtoB = The log2 ratio of the causal model fit over the fit of all other possible models (see Methods). A positive causal score≥1 indicates that the eSNP is predicted to be upstream and causal for the module 9 eigengene.

The most significant SNP regulating *Sfrp1* expression was rs33030926 (P = 5.0×10^−11^) located at 24.587852 Mbp. This SNP was also the most significantly associated with the M9 eigengene (P = 9.0×10^−6^). Rs33030926 is located 27.7 Kbp downstream of the 3′ end of *Sfrp1*. We hypothesized that rs33030926 (or the causal variant linked to rs33030926) regulates *Sfrp1* expression, which in turn influences the co-expression of M9 genes. To test this hypothesis, we used the Network Edge Orienting (NEO) causality modeling R package [37]. NEO is statistical approach used to determine the relationship between genetic variation and two traits. In our case we wanted to determine if rs33030926 affected *Sfrp1* expression and if this in turn perturb the M9. NEO was used to orient the relationships between rs33030926, each of the 15 candidate regulators and the M9 eigengene by determining if the data best fit a causal model (rs33030926→candidate eQTL→M9 eigengene) or one of four competing models that could be used to explain the data. The causal model was the best fit for the *Sfrp1* expression data with a causal score (LEO.nb.AtoB) of 2.80 (Table 3). The LEO.nb.AtoB scores for the other 14 candidate eQTLs were negative (Table 3).

To further characterize the effect of *Sfrp1* on the bone network we stratified mice by rs33030926 genotype and calculated the percent difference in transcript level in *Sfrp1* and all M9 probes. For *Sfrp1*, there was a 10.1% increase in transcript levels in strains (N = 52) homozygous for the rs33030926 “C” allele, relative to strains (N = 44) homozygous for the rs33030926 “T” allele. Similarly, its effect on M9 gene expression was subtle. The max percent difference in expression between rs33030926 genotypes for M9 probes was 27.2% with a mean of 7.4% (Table 4). We conclude that *Sfrp1* induces strong correlations between M9 genes through the subtle coordinate regulation of their expression.

**Table 4:** *Sfrp1* knockdown in primary calvarial osteoblasts preferentially alters the expression of module 9 genes.

Module	ME Chr. 8 SNP r^a^	P	HMDP Diff (%)^b^	P	SiRNA Diff (%)^c^	P	HMDP SiRNA Diff r^d^	P
1	−0.21	4.5×10^−2^	3.5	**<1×10^−4^**	5.1	0.99	0.21	**1.4×10^−4^**
2	0.08	0.45	2.4	0.88	5.5	0.52	0.02	0.41
3	−0.09	0.41	0.6	1.0	3.8	1.0	0.14	6.0×10^−2^
4	−0.28	5.9×10^−3^	2.8	**<1.0×10^−4^**	5.6	0.39	0.07	0.45
5	−0.03	0.78	1.1	1.0	5.5	0.50	0.24	**1.2×10^−5^**
6	0.32	**1.4×10^−3^**	4.6	**<1.0×10^−4^**	6.6	3.4×10^−2^	0.16	**7.8×10^−5^**
7	−0.07	0.51	1.6	1.0	4.1	1.0	0.10	0.19
8	−0.02	0.83	1.3	1.0	4.7	0.93	0.01	0.89
9	0.44	**9.2×10^−6^**	7.4	**<1.0×10^−4^**	8.1	**1.1×10^−3^**	0.32	**4.8×10^−8^**
10	−0.14	0.17	1.8	1.0	6.4	0.11	0.03	0.80
11	0.12	0.25	0.6	1.0	4.8	0.82	0.09	0.30
12	0.13	0.21	1.6	1.0	3.9	1.0	0.10	0.20
13	−0.07	0.53	1.5	1.0	4.5	0.96	0.33	3.0×10^−2^
14	0.01	0.91	1.2	1.0	5.1	0.63	0.03	0.81
15	−0.04	0.68	0.8	1.0	3.8	0.91	−0.35	0.29
16	−0.25	1.3×10^−2^	2.7	0.24	5.5	0.40	−0.21	0.79
17	0.17	9.8×10^−2^	3.6	2.7×10^−3^	5.8	0.29	0.11	0.39
18	0.11	0.27	3.8	**1.0×10^−3^**	6.6	0.11	−0.10	0.53
19	−0.22	3.3×10^−2^	3.2	3.8×10^−2^	6.2	0.20	0.01	1.0
20	0.17	9.5×10^−2^	2.7	0.29	6.2	0.15	0.23	0.20
21	0.08	0.44	1.5	1.0	76.1	**<1.0×10^−4^**	0.02	0.93

a ME Chr.8 SNP r = correlation between module eigengene and the chr. 8 SNP (rs33030926) associated with module 9 and Sfrp1 expression.

b HMDP Diff = mean % difference in module gene expression stratified by rs33030926 genotype ((genotype ‘TT’-genotype ‘CC’)/genotype ‘TT’ *100).

c SiRNA Diff = mean % difference in module gene expression in as a function of *Sfrp1* siRNA knockdown ((*Sfrp1* siRNA-Scrambled Control)/*Sfrp1* siRNA*100).

d HMDP SiRNA = Pearson correlation between HMDP Diff and SiRNA Diff within each module.

All P-values in bold are significant at P≤0.002 (Bonferroni corrected for the number of modules tested).

To add additional support for the causal role of *Sfrp1*, RNAi was used to knockdown *Sfrp1* expression in pcOBs. At four days post differentiation, the expression of *Sfrp1* was measured using qPCR and network-wide gene expression using microarrays. In cells transfected with a siRNA targeting *Sfrp1*, its expression was reduced to 44% (P = 0.001) of the level seen in cells transfected with the scrambled control. Similar to the *in vivo* data in the HMDP, the knockdown of *Sfrp1* early in differentiation (four days post-differentiation) exerted only minor perturbations in network gene expression. Only a small number of the network genes were classified as differentially expressed (FDR<0.05). However, significant (P<0.002) mean differences in expression (difference in expression between pcOBs transfected with *Sfrp1* siRNA and the scrambled siRNA) were observed for modules 9 and 21. In addition, the M9 was the only module with a significant mean percent difference in module gene expression in which there was a significant correlation (r = 0.32, P = 4.8×10^−8^) between the mean percent difference in the HMDP and in response to *Sfrp1* knockdown, indicating that the same M9 genes that were perturbed in the HMDP were also altered due to *Sfrp1* knockdown (Table 4). Our *in vitro* experiments are consistent with *Sfrp1* regulating the coordinate expression of the M9. Together, with the systems genetics analysis from the HMDP, these data identify *Sfrp1* as a regulator of the M9.

### The M9 is associated with BMD in the HMDP

**Figure 7:**
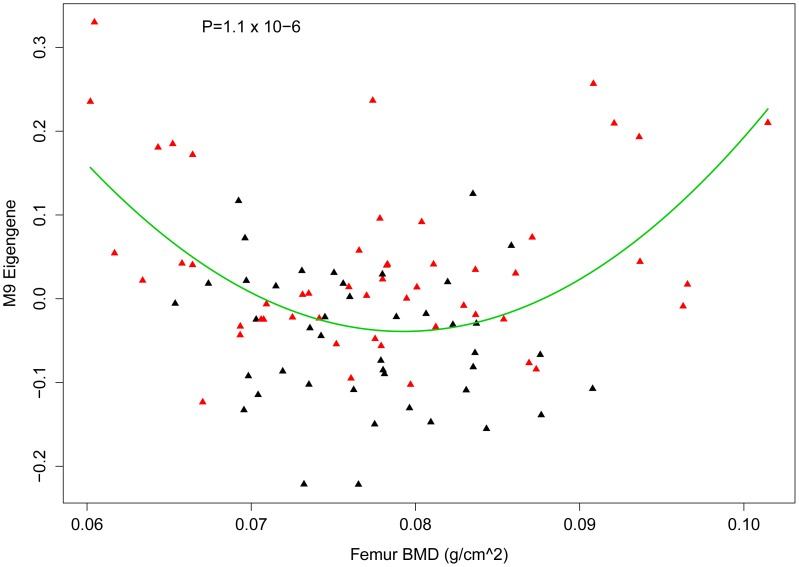
Nonlinear association between the M9 eigengene and BMD. Plot showing the relationship between the M9 eigengene and femur BMD in the HMDP. A quadratic model (M9 eigengene = BMD+BMD∧2) significantly (P = 1.1×10^−6^) fit the data. The quadratic line is shown in green. Each diamond represents a strain and those in red are homozygous ‘TT’ and those in black are homozygous ‘CC’ at rs3303926 SNP that regulates *Sfrp1* expression and the M9 eigengene.

If M9 behavior in the HMDP is reflective of osteoblast/osteocyte activity then we would expect that it would be associated with changes in bone mass in the HMDP strains. We previously measured BMD in all HMDP strains [27]. To assess its relationship with BMD, we determined the correlation between the M9 eigengene and BMD. The M9 eigengene was not linearly correlated (r = −0.03; P = 0.71) with femoral BMD, however, as shown in Figure 7 there was a U-shaped relationship between the two. Based on this observation we fit a quadratic model (M9 eigengene = BMD+BMD∧2) to the data (Figure 7). The quadratic model was a highly significant fit (P = 1.1×10^−6^). A shown in Figure 7, strains with the highest M9 had either low or high BMD. As would be expected based on the observation that rs33030926 regulates *Sfrp1* and the M9 eigengene, all the strains with high expression of the M9 eigengene and the lowest and highest BMD were ‘TT’ homozygotes (Figure 7). Similar patterns were observed for total body and spinal BMD (data not shown). Importantly, these data provide additional evidence of the biological relevance of the M9. It also suggests that the M9 reflects the complex, and often contradictory, role of osteoblast-lineage cells in bone homeostasis.

### M9 is enriched for genes implicated in human BMD GWA studies

To evaluate the potential relevance of the M9 to BMD in humans we determined if it contained genes that have been implicated in the regulation of BMD through human GWA studies. We used information from the largest GWA analysis for BMD performed to date. This study meta-analyzed data from 17 BMD GWA studies (N = ∼32 K in the discovery phase and N = ∼50 K in the replication phase) [38]. In this meta-analysis, a total of 64 independent SNPs reached genome-wide significance implicating 56 regions and 61 unique genes (these genes were the closest to the most significant independent GWA SNPs). We were able to identify a mouse homolog for 57 of the 61 genes (93%) and 39 were located within one of the 21 network modules (Table 5). Of these, five (8.7% of the total) (*Lrp5, Tnfrsf11b, Wnt4, Gpr177 and Sp7*) were members of the M9. The probability of identifying five M9 genes among 57 randomly chosen genes from the network was P = 5.0×10^−4^. After a Bonferroni correction for the 21 modules, the M9 was the only module to demonstrate this enrichment. These data indicate the M9 is enriched for genes that have been implicated in the regulation of BMD in humans.

**Table 5:** Module 9 is enriched in homologs of human genes implicated in the regulation of BMD through genome-wide association studies.

Module	No. Unique Module Genes^a^	No. BMD GWAS homologs^b^	Enrichment P-value^c^	Genes
1	2504	4	0.70	*Anapc1, Sox6, Mpp7, Arhgap1*
2	2204	5	0.39	*Mef2c, Mapt, Cdkal1, Cyld, AW548124*
3	1715	4	0.40	*Sox9, Rps6ka5, AI597468, Smg6*
4	981	0	1.0	
5	1077	3	0.34	*Ctnnb1, Jag1, Lin7c*
6	910	7	0.02	*Hoxc6, Kcnma1, Spnb2, Sost, Wnt16, Mepe, A430107O13Rik*
7	615	4	0.13	*Mbl2, Slc25a13, Insig2, Cpn1*
8	425	1	0.55	*Mark3*
9	354	5	**5×10^−4^**	*Lrp5, Tnfrsf11b, Wnt4, Gpr177, Sp7*
10	301	1	0.43	*Klhdc5*
11	278	1	0.40	*BC030867*
12	219	0	1.0	
13	199	0	1.0	
14	171	1	0.27	*Abcf2*
15	96	0	1.0	
16	94	0	1.0	
17	76	1	0.14	*Tnfrsf11a*
18	68	1	0.12	*Rspo3*
19	74	0	1.0	
20	69	1	0.12	*Dhh*
21	54	0	1.0	

a Number of unique genes in each module.

b Number of human homologs implicated in the regulation of BMD in [38].

c P-value for the enrichment of human BMD genes.

## Discussion

In this study, we generated a co-expression network for bone that consisted of 21 “modules”, each of which contained genes that shared similar expression patterns and were enriched for functionally similar genes. We then focused on one module, the M9, which was predicted to be specific for cells of the osteoblast-lineage. We demonstrated that the perturbation of M9 hub genes altered osteoblast proliferation and differentiation and for one hub, *Maged1*, BMD *in vivo*. Additionally, we discovered that an *Sfrp1* local eQTL was the key driver of M9 gene expression, that the M9 was associated with BMD in the HMDP and was enriched for the homologs of genes implicated in the regulation of BMD through human GWA studies.

Traditional genetic and molecular approaches are powerful tools for dissecting cellular function, however, reductionist techniques may not be able to capture the overall organization of cellular interactions. Systems genetics is an approach that can provide an unbiased and more comprehensive view of not only the genes involved in cell function, but also key gene-gene interactions. By using the hundreds of thousands of genetic perturbations that exist in the HMDP to identify correlational patterns between genes on a genome-wide scale we were able to discover a core group of 354 genes that are highly co-expressed and function together in a network. We believe that this network acts to propagate or modulate major osteoblastic stimuli, such as *Wnt* signaling. Importantly, the M9 represents a wealth of information that can be mined in future experiments to increase our understanding of the genes and interactions that are critical for proper osteoblast-lineage function.

The use of network analysis provided a number of unique advantages. First, WGCNA gave us the opportunity to group genes into modules based on their *in vivo* patterns of expression in whole bone and then determine which module was the most relevant to cells of the osteoblast-lineage. Second, in a traditional differential expression analysis across strains, only a small percentage of M9 genes would have been identified as differentially expressed and thus, potentially important in bone. Third, the discovery that M9 connectivity was highly correlated with GS could have only been made via network analysis. Lastly, integrating network analysis and GWA identified *Sfrp1* as a regulator of the M9. Although *Sfrp1* is known to play an important role in the osteoblast lineage, our results have identified an entire network of genes that are novel downstream targets of *Sfrp1*.

A number of recent works have identified “module quantitative trait loci (mQTL)” (as examples [35], [39], [40]). Here, we identified *Sfrp1* as the gene and its local eQTL as the mechanism underlying the mQTL regulating the M9 eigengene. This represents one of the first successful attempts at identifying the molecular basis of an mQTL. This was possible due to the ability to perform high-resolution genome-wide association in the HMDP and the tools of systems genetics. This study highlights the advantages of disentangling the genetics of co-expression module regulation using a high-resolution genetic reference population such as the HMDP.

We observed that the M9 eigengene was inversely correlated with BMD in low bone mass mice and positively correlated with BMD in high bone mass mice. This nonlinear association is likely due the complex roles of osteoblast-lineage cells in bone [1]. Osteoblasts directly control bone formation and secrete Osteoprotegerin, a strong inhibitor of bone-resorbing osteoclasts [1]. Moreover, pre-osteoblasts and recently osteocytes, have been shown to secrete RANKL, which promotes osteoclastogenesis and bone resorption [8], [9], [41]. Therefore, M9 “activity” likely represents a balance between osteoblast-mediated bone formation and osteoblast/osteocyte-directed bone resorption. It is possible that the differential effect of high M9 activity in the HMDP is due to differences cell composition (e.g. differences in the relative numbers of osteoprogenitors, mature osteoblasts and osteocytes) or other factors that are determined by genetic background. Consistent with the role of genetic background, many of the low BMD HMDP strains with high M9 eigengene expression belonged to the AXB recombinant inbred set. More detailed phenotyping of strains with high M9 expression and low or high BMD will be needed to clarify the difference. At any rate, the association between M9 and BMD indicates that it reflects physiologically relevant differences in the activities of osteoblast-lineage cells.

We identified a strong correlation between M9 connectivity (kme) and GS. This finding is important since it suggests that not only are M9 genes important, but the topology of the M9 network is also important for the function of osteoblast-lineage cells. This finding allowed us to use kme information to prioritize genes for validation. Because the most highly connected genes were the most correlated with GS, we choose the top two hubs for further investigation. Many of the top ten hubs are known to function in osteoblasts (Table 2); however, the top two, *Maged1* and *Pard6g*, have not been shown to directly participate in osteoblast proliferation, differentiation or mineralization. Using RNA interference we demonstrated that both genes play a role in osteoblast activity.

The siRNA knockdown of *Maged1* increased the proliferation of primary calvarial osteoblasts. It also increased the early expression of alkaline phosphatase, a marker of maturing osteoblasts. Surprisingly though, we found that it decreased mineralized nodule formation. *Maged1* is a transcriptional co-activator involved in a wide-array of cellular processes such as the regulation of myogenic differentiation, circadian rhythms, sexual behavior and obesity, to name a few [30], [31], [42]. *Maged1* has been shown to bind to the homeodomain protein DLX5 and is required for its transcriptional function [32]. Consistent with *Maged1* affecting osteoblast function through DLX5, mineralized nodule formation in osteoblasts from *Dlx5^−/−^* knockout mice is also lower. However, proliferation in *Dlx5^−/−^* osteoblasts is also decreased in contrast to the increase in proliferation we observed when *Maged1* is knocked-down. The effect of *Maged1* on proliferation in osteoblasts, however, is consistent with the observations that it inhibits proliferation in other cell-types [43], [44]. This suggests the *Maged1* may have effects on osteoblast function independent of DLX5 activity. The increase in alkaline phosphatase and *Sp7* expression at 4 days post-differentiation (both markers of osteoblast differentiation) is hard to reconcile with the decreased mineralized nodule formation at 14 days post-differentiation. It is worth noting that *Sp7* and *Akp2* were the only osteoblast markers that were increased, which suggests that *Maged1* knockdown may selectively result in increased *Sp7* and *Akp2* expression without inducing the complete differentiation cascade. However, as suggested above it could also reflect diverse roles for *Maged1* in the osteoblast. An alternative explanation is that the conflicting early increase and late decrease in osteoblast differentiation is a result of the transient nature of *Maged1* knockdown with siRNA. Most importantly, however, we demonstrate that Maged1 deficiency in vivo results in decreased BMD. This is consistent with decreased mineralized nodule formation in vitro. It is also consistent with Maged1 mediating its effects on osteoblast function through DLX5, as *Dlx5* deficient mice also have decreased bone mass [33]. Further work is needed to define the precise role of *Maged1* in osteoblasts and how this translates into lower bone mass.

The Par6 (partition defective) family of proteins was first identified in C. elegans and Drosophila as proteins required to establish cell polarity [45]. There are three homologues of Par6 in mammals, PARD6A, 6B and 6G [46]. The siRNA knockdown of *Pard6g* decreased both osteoblast proliferation and differentiation. It is possible that these effects of *Pard6g* are due strictly to the fact that cell polarity is an essential cellular process and are not necessarily reflective of Pard6g function in osteoblasts. However, we feel this is unlikely given its membership in the M9 and the fact that its expression is high in osteoblasts and its expression differs as a function of osteoblast differentiation. In addition, Par6 has recently been shown to be involved in skeletogenesis and biomineralization in the sea urchin [47]. Although more work is needed it is tempting to speculate that *Pard6g* is involved in *Wnt* signaling. Non-canonical Wnt signaling is a major regulator of cell polarity and the M9 contains non-canonical Wnts such as *Wnt4* (also a gene associated with BMD in humans) [48].

The *Wnt* signaling pathway is a major pro-osteoblast stimulus [10]. In osteoblasts, *Wnts* bind frizzled (*Fzd*) receptors and their co-receptors (LRP5 and LRP6) and induce the stabilization and translocation of ß-catenin to the nucleus [10]. *Sfrp1* antagonizes *Wnt* signaling by interfering with the interaction between *Wnts* and *Fzd* receptors [49]. *Sfrp1* knockout mice are resistant to age-related decreases in trabecular bone mass and display reduced osteoblast/osteocyte apoptosis and increased osteoblast proliferation and differentiation [50]. Conversely, *Sfrp1* transgenic mice have decreased trabecular and cortical BMD and decreased osteoblast proliferation and differentiation [25]. Using genome-wide association we identified *Sfrp1* as a regulator of M9. Based on its known role in cells of the osteoblast-lineage, its discovery as a major regulator of the M9 is consistent with M9 representing a core network operative in osteoblasts/osteocytes.

There has been interest in developing therapeutics targeting *Wnt* signaling in general and *Sfrp1* specifically. In fact, Bodine *et al.* demonstrated that piperidinyl diphenylsulfone sulfonamide could bind and inhibit the activity of SFRP1 [51], [52]. However, there is concern that *Sfrp1* is a poor drug target based on its broad tissue expression and the link between *Wnt* signaling and various cancers [26]. Given that *Sfrp1* regulates M9 gene expression it is likely that many M9 genes are downstream targets of *Sfrp1* and more generally *Wnt* signaling. This notion is supported by the observation that the perturbation of *Maged1* and *Pard6g* expression resulted in similar effects on pcOBs as did *Sfrp1* alteration [25]. Targeting M9 genes could promote increased bone formation in a more bone-specific manner.

Recent studies have demonstrated that co-expression modules can be conserved across species; therefore, it is of significant interest to know if the human homologs of M9 genes function in a similar network. We will directly investigate this in future studies, however, the fact that conserved pathways (primarily *Wnt* signaling) are represented in the M9 suggests that the majority of M9 genes will also play a role in the function of osteoblast-lineage cells in humans. Additionally, the fact that nearly 10% of genes implicated in the most comprehensive BMD GWA meta-analysis performed to date are members of the M9 further support the notion that the M9 is relevant to the regulation of human BMD. It is worth noting that 4 of the 5 M9 human BMD genes are involved directly in *Wnt* signaling, which again suggests that this signaling pathway is a major component of the M9.

Our approach is not without limitations. One possible limitation, which also may have been an advantage, was the generation of expression data from bone tissue. An alternative approach would have been to profile isolated cell populations. This may have been more informative since it is clear that the different cells in the osteoblast-lineage perform distinct and often contradictory functions. On the other hand, the profiling of bone cells in their native complex cellular milieu may have resulted in expression profiles that were more representative of their true *in vivo* state. At any rate, we do believe that it will be informative in future studies to repeat this analysis using isolated cell populations as this may remove some of the noise associated with averaging expression across multiple cell-types. A second limitation was our use of siRNA in primary calvarial osteoblasts to validate the role of *Sfrp1*, *Maged1* and *Pard6g*. In terms of *Sfrp1*, we perturbed its expression *in vitro* at one time point in osteoblasts isolated from neonatal mice. Although this system allowed us to test the hypothesis that *Sfrp1* preferentially modulated the expression of M9 genes, it did not fully recapitulate the effects of *Sfrp1* expression differences in the HMDP. It is known that *Sfrp1* has many effects on osteoprogenitors, mature osteoblasts and osteocytes [50]. Thus, it would have been more ideal to test the effect of *Sfrp1* perturbation *in vivo*, which will be the focus of future investigations. The same is true for *Maged1* and *Pard6g*. Although we clearly demonstrate their involvement in osteoblast proliferation and differentiation, it is possible that we missed many aspects of their function by focusing on just two stages in the complicated life-cycle of an osteoblast-lineage cell. This is especially true in the case of *Maged1* were we observed that its reduction increased certain aspects of early osteoblast differentiation, but later decreased mineralized nodule formation.

In summary, we have used a systems genetics approach consisting of co-expression network analysis, eQTL analysis, genome-wide association and causality modeling in a powerful mouse genetic reference population to identify a module (M9) of co-expressed genes that play an important role in the function of osteoblast-lineage cells. These data improve our understanding of the gene networks important for osteoblast function and demonstrates the ability of systems genetics to unravel gene networks involved in complex cellular processes.

## Materials and Methods

### Ethics statement

The Institutional Care and Use Committee (IACUC) at the University of California, Los Angeles approved the animal protocol for the HMDP. The animal protocol for the isolation of primary calvarial osteoblasts was approved by the University of Virginia IACUC. The manipulations of *Maged1-*deficient mice has been approved by the local ethics committee of the University of Namur and follow the European legislation.

### Bone expression profiles and BMD in the Hybrid Mouse Diversity Panel

Data from Hybrid Mouse Diversity Panel (HMDP) were generated from 16-week old male mice from 96 inbred strains. More details regarding the population can be found in [27], [28]. RNA isolation and Illumina microarray processing for bone tissue samples from the HMDP are described in [26]. The expression data are available from the NCBI Gene Expression Omnibus (GEO) database (GSE27483). The quantification of femoral, spinal and total BMD in the HMDP has been described in [27].

### Analyzing BMD in *Maged1*-deficient mice

The *Maged1*-deficient mice and control littermates used in this study have already been described [53]. Experiments were done with male mice aged 18 weeks backcrossed for >10 generations in the C57Bl/6J genetic background. Body composition and BMD was measured using a dual-energy X-ray absorptiometry (DEXA) scanner (Lunar PIXImus2; GE Healthcare). All scans were analyzed using the PIXImus2 software (version 2.10). For the calculation of total body BMD the skull was excluded from the analysis.

### Weighted gene co-expression network analysis

Network analysis was performed using the WGCNA R package [54]. All 45,759 array probes were used to construct the bone network. We did not collapse multiple probes per genes down to a single probe representing each gene since many of the seemingly redundant probes actually recognize alternatively spliced isoforms. We also did not have to worry that the inclusion of probes that were not expressed would add noise, since the vast majority of such probes would not be expected to exhibit biologically meaningful correlations with a large number of other transcripts. The approach also allowed for the inclusion of probes that are truly expressed, but at a level that may not have exceeded a particular “expressed/not expressed” threshold. To generate the co-expression network, we first calculated Pearson correlation coefficients for all gene-gene comparisons across all microarray samples. The matrix of correlations was then converted to an adjacency matrix of connection strengths. The adjacencies were defined as 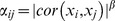 where 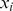 and 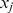 are the 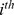 and 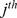 gene expression traits. The power 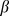 was selected using the scale-free topology criterion previously outlined by Zhang and Horvath [55]. In this study a 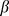 = 8 was used. Modules were defined as sets of genes with high topological overlap [21]. The topological overlap measure (TOM) between the 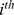 and 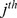 gene expression traits was taken as 
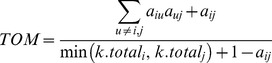
, where 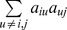 denotes the number of nodes to which both 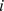 and 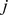 are connected, and 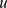 indexes the nodes of the network. A TOM-based dissimilarity measure 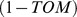 was used for hierarchical clustering. Gene modules corresponded to the branches of the resulting dendrogram and were precisely defined using the “Dynamic Hybrid” branch cutting algorithm [56]. A principal component analysis was used to generate a vector of values (first principal component) that summarized or were the most representative of each modules expression. Intramodular connectivity (kme) was defined as the correlation between a gene's expression and its module eigengene. Highly similar modules were identified by clustering and merged together. Network depictions were constructed using Cytoscape [57].

### Module characterization

The gene content of each module was characterized using the DAVID gene enrichment analysis tool [58], [59]. Gene Significance (GS) for each network gene was defined as the absolute value of its correlation with the eigengene of a set of nine osteoblast/osteocyte marker genes identified from the literature. Module Significance (MS) was calculated as the mean GS for each module. Significance of the MS was determined for each module by randomly selecting x GS scores from the set of 13,579 network GS scores; where _x_ is equal to the number of genes in that module. The mean for each set was then calculated and this was repeated 10,000 times. The true MS score was then compared to the distribution of random mean GS scores and P-values were calculated by counting the number of random mean GS scores that were greater than the true MS score divided by 10,000. An MS score with a P<0.002 (Bonferroni adjusted for the 21 modules tested) was deemed significant. To determine the expression patterns of network genes in bone and bone cells microarray data on primary osteoclasts, primary osteoblasts, whole bone and bone marrow were downloaded from GEO (GSE11339 and GSE10246). The samples were derived from C57BL6/J mice in triplicate. The osteoblast samples were comprised of three different time-points (5, 14 and 21 days of differentiation) assayed in triplicate. For each module the log2 fold expression of its genes in osteoblasts (highest of the three time-points) were compared to the other samples. Statistical significance of the increase in expression in osteoblasts was determined as described above for GS and MS.

### EMMA and eQTL hotspot detection

EMMA and its application to HMDP data has been described in [27], [28], [36], [60]. EQTL hotspots for the M9 were identified by performing genome-wide association for the expression of all 13,759 network probes using EMMA. SNPs were clustered into 531, 5-Mbp bins across the genome and for each network probe, the minimum association p-value was recorded for each bin and the number of probes with p-values that exceeded −logP≥4 were counted. Enrichment P-values were then assigned to each bin using a Fisher's exact test to compare the frequency of significant associations for M9 probes relative to all other network probes. Bins with P<0.05/531 = 9.4×10^−5^ were deemed significant.

### Causality modeling using network edge orienting (NEO)

Causality modeling was performed as described in [61], [62]. Briefly, NEO is an R function designed to orient the relationships between genetic markers, gene expression traits and clinical traits [37]. NEO utilizes the fact that all cellular information begins with DNA and therefore, the many possible relationships that can exist between DNA variation, gene expression and clinical traits can be distilled to three. The three relationships (or models) are: 1) causal – flow of information goes from DNA to gene to BMD (gene's expression is causing the change in the trait); 2) reactive – flow of information goes from DNA to BMD to gene (gene's expression is reacting to the change in the trait) and 3) independent – DNA variation affects both traits independently. NEO uses structural equation modeling to estimate the probabilities for each of the three relationships. The log10 ratio of the causal model probability relative to the next best model probability (of the two remaining) is then calculated. This ratio (referred to as the LEO next best or LEO.NB score) quantifies the relative likelihood that a gene's expression is causal for a trait such as BMD. Simulation studies have demonstrated that single marker LEO.NB scores above 1.0 are highly suggestive of causal relationships [37].

### Analysis of human GWA data

The mouse homologs for human genes nearest the most significant SNP for all genome-wide significant associations identified in [38] were identified. A clear homolog was identified for 57 associations. The module membership for each of the 57 genes was then determined. Enrichment p-values for each module were calucated by randomly selecting×genes, where x is the number of genes in a given module, out of the 30,264 unique genes used to generate the network. This was repeated 10,000 times. The number of randomly selected genes that overlapped the GWA set in each random selection was then recorded. The enrichment P-value was calculated as the number of times (out of 10,000) the overlap equaled or exceeded the actual number observed for each module. An enrichment P-value corrected for the 21 modules (0.05/21 = 0.0023) was deemed significant.

### Isolation of pcOBs

Three to nine day old neonates from C57BL/6J breeding pairs (obtained from Jackson Laboratory, Bar Harbor, ME) were euthanized by CO_2_ inhalation followed by decapitation. The heads were sprayed with 70% ethanol and placed in sterile cold DPBS (Gibco). Using sterile instruments, the skin was removed from the skull, calvariae were removed and placed into sterile cold DPBS (Gibco). Harvested calvariae were then placed in sterile digestion solution (0.05% Trypsin-EDTA, 1.5 U/ml Collagenase P, MEM Alpha) and incubated at 37°C, with 120 rpm shaking for 15 minutes. Four digests were performed, the first being discarded. For the remaining three digests an equal volume of sterile plating media (DMEM, 10% heat-inactivated FBS, 100 U/ml penicillin, 100 ug/ml streptomycin) was added to each immediately following its collection and stored on ice. The fractions were combined, filtered through a 100 uM sterile vacuum filtration tube and counted.

### Differentiation of pcOBs

Cells isolated from 4–6 independent groups of 3–9 day old neonates were plated into 6 well plates at 300,000 cells/2 ml sterile plating media (DMEM, 10% heat-inactivated FBS, 100 U/ml penicillin, 100 ug/ml streptomycin) per well. After 24 hours, confluent cells (Day 0) were washed 1× with DPBS (Gibco) and placed in sterile differentiation media (DMEM, 10% FBS, 100 U/ml penicillin, 100 ug/ml streptomycin, 0.1 M ascorbic acid, 1 M B-glycerophosphate). Every 48 hours thereafter cells were washed one time with DPBS (Gibco) and differentiation media replaced.

### siRNA transfection of pcOBs

PcOBs were plated at 150,000 cells/2 ml plating media per well. Transfections were performed using Lipofectamine 2000 Reagent (Invitrogen) according to manufacturer's directions. A total of three Stealth Select RNAi siRNAs (Invitrogen) per gene were first tested using three different concentrations (0.2 nM, 2.0 nM or 10 nM). Knockdown of the target gene was tested at 48 hours using qPCR in undifferentiated pcOBs (see below). All siRNAs demonstrated the most effective knockdown at 10 nM. The two most effective siRNAs for each gene were used for all downstream experiments with the exception of *Sfrp1* and only one of the three provided more than 50% knockdown. The sense strand of the duplex siRNA sequences were as follows: *Sfrp1* - MSS277026; CCGAGAUGCUCAAAUGUGACAAGUU; *Maged1* – MSS294723; GCAAGGUUAAUAACUUGAAUGUGGA and MSS235163; UCAGAACGUGGAGUCCCGGACUAUA; *Pard6g* MSS234948; GCAACGGCAGCAUCCACAGAUUUCU and MSS234949; CAUAAGUCUCAGACCCUACGCUUCU. The Stealth RNAi Negative Control Duplex (Invitrogen) was used as a scrambled control. As a control, we also demonstrate in File S3 that knockdown of *Kdelr3* (a gene expressed in primary calvarial osteoblasts) has no effect on mineralized nodule formation, providing additional support that the effects of target gene siRNA are due specific to the knockdown of *Maged1* and *Pard6g* (File S3). At 24 hours post-transfection, cells were trypsinized (0.25% Trypsin-EDTA) and re-plated in a 12 well plate. The following day cells reached 100% confluency (Day 0) and were washed 2× with sterile DPBS (Gibco) and placed in sterile differentiation media (DMEM, 10% FBS, 100 U/ml penicillin, 100 ug/ml streptomycin, 0.1 M ascorbic acid, 1 M B-glycerophosphate). Every 48 hours thereafter cells were washed 1× with DPBS (Gibco) and differentiation media replaced.

### Protein isolation and Western blots

Protein was extracted from pcOBs in 10% NP40 detergent containing protease inhibitors (Thermo Scientific). The extracts were separated on 12% NativePAGE™ Novex Bis-Tris Gels (Life Technologies) and transferred to PVDF membranes. Antibodies were obtained from Santa Cruz Biotechnology (PARD6G) and Milipore (MAGED1). Bound antibodies were visualized using the Western Lightning Plus-ECL (Perkin-Elmer). ImageJ (NIH) was used to quantify individual bands by normalizing the density of the target band (MAGED1 or PARD6G) by the density of the ß-ACTIN band for each sample.

### Analysis of gene expression

Total RNA was isolated using RNeasy Mini-Kit (Qiagen) according to manufacturer's instructions followed by genomic DNA decontamination using DNA-free kit (Applied Biosystems) according to manufacturer's directions. cDNA was synthesized using the High-Capacity cDNA Reverse Transcription kit (Applied Biosystems) according to manufacturer's instructions using C1000 Thermal Cycler (Bio-Rad). Quantitative real-time PCR (qPCR) was performed on 50 ng cDNA template, 10 uM each forward and reverse primer, and SensiMix Plus SYBR kit (Quantace) according to manufacturer's directions in 20 ul total volume using an ABI 7900 thermocycler. The following primer sets were used (all sequences 5′-3′): Maged1–F, AGATGGCTCCCAGACTCAGA; Maged1–R, CCTTTGATCCCCACTGTTGT; Pard6g–F, TGACGACAACTTCTGCAAGG; Pard6g–R, GCTCCGAAGCTGTAATGGTC; Sfrp1-F, TACCACGGAAGCCTCTAAGC; Runx2-F, ACAGTCCCAACTTCCTGTGC; Runx2-R, CACAGTCCCATCTGGTACCTC; Sfrp1-R, TCGCTTGCACAGAGATGTTC; Sp7–F, TGCCCCAACTGTCAGGAG; Sp7–R, GATGTGGCGGCTGTGAAT; Akp2–F, CCTTGAAAAATGCCCTGAAA; Akp2–R, TTACTGTGGAGACGCCCATA; Ibsp-F, GAGGAGACTTCAAACGAAGAGG; Ibsp-R, ACACCCGAGAGTGTGGAAAG; Col1a1-F, CCCAAGGAAAAGAAGCACGTC; Col1a1-R, AGGTCAGCTGGATAGCGACATC; Bglap2–F, GAACAGACAAGTCCCACACAGC; Bglap2–R, AGAGACAGAGCGCAGCCAG; 36B4-F, ACTGAGATTCGGGATATGCTGT; 36B4-R, TCCTAGACCAGTGTTCTGAGCTG. Relative quantification was determined by the 2(−Delta Delta CT)) method using the 36B4 gene as reference gene [63]. The results were obtained from N = 4 independent experiments.

### Microarray analysis of transfected pcOBs

Microarray expression profiles were generated using the MouseWG-8v2 BeadChips (Illumina, San Diego, CA). Briefly, biotin-16-UTP labeled cRNA was synthesized using the Illumina TotalPrep RNA Amplification Kit (Ambion, Austin, TX). A total of 850 ng of cRNA was then hybridized to the Illumina BeadChips. Microarrays were scanned using the Illumina iScan system and background corrected signal intensities were extracted using the GenomeStudio software (Illumina). The lumi R package was used to transform the data using a Variance Stabilizing Transformation (VST) and normalized using quantile normalization [64].

### Osteoblast proliferation and differentiation assays

Proliferation was measured in pcOBs by plating cells at a density of 2000 cells/dish in 96 wells, treating as indicated and proliferation rate was determined using the BrdU ELISA assay (Roche). The results were obtained from N = 6 independent experiments. Quantitative analysis of soluble alkaline phosphatase activity in cell extracts was performed using a colorimetric kit (AnaSpec) that measures the conversion of p-nitrophenyl phosphate to p-nitrophenol according to the manufacturer's instructions. Alkaline phosphatase activity was normalized to protein concentration. Protein levels were determined using using the bicinchoninic acid (BCA) assay according to the manufacturer's instructions (Pierce). Mineralized nodule formation was measured by staining cultures at 12 days post-differentiation with Alizarin Red (40 mM) (pH 5.6). The stained cells were imaged and nodule number was measured using ImageJ (NIH). Alizarin Red was quantified by destaining cultures with 10% Acetic Acid and determining the optical density (405 nM) of the resulting solution. The results were obtained from N = 4 independent experiments.

## Supporting Information

File S1 Module assignments for all network probes. This file contains the module assignments and annotations for each of the 13,579 microarray probes contained in the bone co-expression network. Columns A–F provide probe annotations. Column G contains the module assignments and columns H-AB contain the correlation between each genes expression and module eigengene for each of the 21 modules. This correlation is the kme for genes within a given module.
(CSV)

File S2 Significant (FDR<0.05) gene ontology enrichments for all 21 modules. This file is the output from DAVID and contains (FDR<0.05) GO and KEGG ontology enrichments for each module. For each module, columns B and C define the enriched term. Column C–K contains, the number of module genes in each category (Count), the percentn of module genes in each category (Percent), the raw enrichment P-value (P-value), gene IDs, the total number of genes in each category (List.Total), the total number of genes in the genome in each category (Pop.Hits) and the total number of genes in the genome used to calculate each categories enrichment (Pop.Total). Columns L, M and N contain P-values adjusted for multiple comparisons using Bonferroni, Benjamini and FDR corrections. More information about the output can be found at (http://david.abcc.ncifcrf.gov/).
(CSV)

File S3 Additional siRNA controls. We knocked down the expression of Kdelr3 (a member of M9 and a gene expressed in osteoblasts) in primary calvarial osteoblasts as described in methods. Its knockdown using two independent siRNAs resulted in >95% knockdown (*P<0.05). Its knockdown did not alter mineralized nodule formation. This confirms activation of the RNA-induced silencing complex (RISC) and that the effects of *Maged1* and *Pard6g* knockdown are not due to an alteration in overall cell function.
(PDF)
