# Supplementary figures and images for: Systems Genetic Analysis of Osteoblast-Lineage Cells
Source: PLoS Genet. 2012 Dec 27;8(12):e1003150. doi: 10.1371/journal.pgen.1003150 (PMC3531492; doi:10.1371/journal.pgen.1003150)

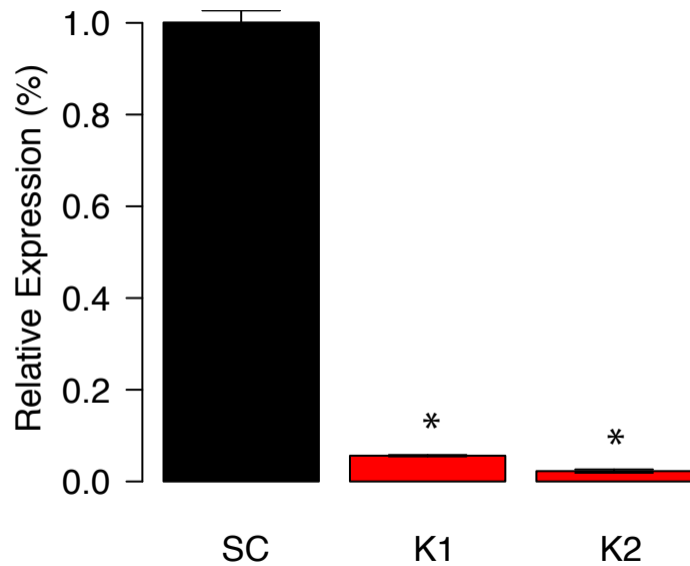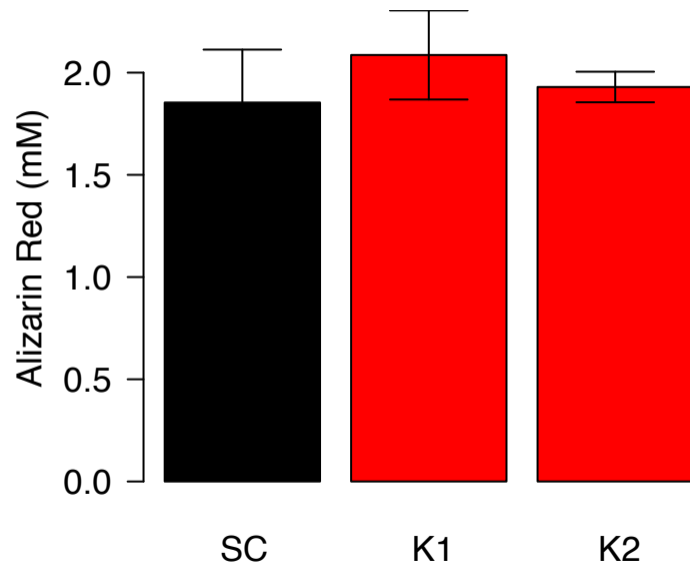

Supplement: File S3 — Additional siRNA controls. We knocked down the expression of Kdelr3 (a member of M9 and a gene expressed in osteoblasts) in primary calvarial osteoblasts as described in methods. Its knockdown using two independent siRNAs resulted in >95% knockdown (*P<0.05). Its knockdown did not alter mineralized nodule formation. This confirms activation of the RNA-induced silencing complex (RISC) and that the effects of Maged1 and Pard6g knockdown are not due to an alteration in overall cell function. (PDF) [file pgen.1003150.s003.pdf]
